# Sr–Pb isotope differences in pre- and post-burial human bone, teeth, and hair keratin: implications for isotope forensics

**DOI:** 10.1007/s00414-023-02976-5

**Published:** 2023-02-23

**Authors:** Lisette M. Kootker, Saskia T. M. Ammer, Daniel J. Wescott, Gareth R. Davies, Hayley L. Mickleburgh

**Affiliations:** 1 Geology & Geochemistry Cluster, Vrije Universiteit Amsterdam, De Boelelaan 1085, 1081 HV Amsterdam, the Netherlands; 2 Co Van Ledden Hulsebosch Centre (CLHC), Science Park 904, 1098 XH Amsterdam, the Netherlands; 3 Forensic Anthropology Centre, Department of Anthropology, Texas State University, 601 University Drive, San Marcos, TX 78666 USA; 4 Amsterdam Centre for Ancient Studies and Archaeology, Faculty of Humanities, University of Amsterdam, P.O. Box 94203, 1090 GE Amsterdam, the Netherlands

**Keywords:** Isotope forensics, Diagenesis, Geographic origin, Human decomposition, Forensic taphonomy, Human tissues

## Abstract

The isotopic signatures of human tissues can provide valuable information on geographic origin for medicolegal investigations involving unidentified persons. It is important to understand the impact of diagenetic processes on isotopic signatures, as alterations could result in incorrect estimation of geographic origin. This study examines alterations in isotope signatures of different tissues of five human body donors studied throughout decomposition at the Forensic Anthropology Research Facility (FARF), San Marcos, TX. Two body donors were buried, two were placed in open pits, and one was first allowed to naturally mummify and then buried. Remains were recovered after a period of 7–34 months. The preplacement and post-recovery Sr–Pb isotope data of scalp hair, bone (iliac and tibia), and tooth enamel and dentine were compared. The hair samples record significant shifts in Sr-Pb isotope compositions, with hair keratin Pb isotope composition shifting towards the Pb signature of local soil samples. Hair keratin Sr isotope compositions were altered by the burial environment and possibly also by the lab sample cleaning method. The spongy iliac bone samples show inconsistencies in the recoverability of the preplacement Sr–Pb isotope signatures. The post-placement signatures of the buried donors show slight elevation over preplacement signatures. The post-placement signatures of donors placed in open pits are significantly elevated. The tibia and dental samples record the most consistent isotopic data with the least alteration. These more densely mineralised elements show good recoverability of the preplacement isotope signatures in burials and open pits and are thus deemed better targets for forensic investigative purposes.

**Supplementary Information:**

The online version contains supplementary material available at 10.1007/s00414-023-02976-5.

## Introduction

While related fields, such as archaeology, have been using stable and radiogenic isotope analysis to investigate geographical origin for decades [1–5], forensic sciences, and forensic anthropology in particular, have only recently started to use these methods for medico-legal purposes [6–8]. Isotope data can aid the identification of unknown individuals by providing information on (A) the geographical origin of individuals and their circumstances during different periods of their lives using the isotope systems of, e.g. oxygen (δ^18^O), hydrogen (δ^2^H), strontium (^87^Sr/^86^Sr), and lead (^20x^Pb/^20x^Pb), and (B) the dietary preferences using the isotope systems of, e.g. carbon (δ^13^C), nitrogen (δ^15^N), and sulphur (δ^34^S) [9]. Notable cases in which isotope analysis has provided accurate information on the whereabouts and life circumstances of unidentified individuals include the “Berlin case” [10], the waterlogged remains in Amsterdam [11, 12], and most recently, the “Burgenland case” [13]. In another prominent example, Meier-Augenstein and Fraser used isotope data to provide investigating officers with crucial information about the previous movements of the victim, as well as a lead to conduct DNA analysis on a presumed child of the victim. This ultimately led to the identification of the victim and aided the apprehension of the perpetrators, known as the “scissor sisters,” who dismembered and mutilated the body [14].

These examples emphasise the benefits and medico-legal significance of analysing different types of tissue and skeletal elements for the purpose of obtaining information on different periods in life. Dental enamel and dentine of the permanent dentition reflect the diet and geolocation of an individual during childhood (birth until ca. 16 years in enamel [15, 16]) until early adolescence (ca. 3–25 years in dentine [15, 16]), while isotope data from hair and nail keratin shows the more recent diet and mobility patterns (weeks to years prior to death, respectively [17–20]. The isotope composition of bone depends on the bone remodelling rate and reflects time ranges from ca. In total, 5–30 years prior to death, even 50 years in some cases (i.e. cranial bones). Bone remodelling depends on, inter alia, bone type (trabecular or cancellous vs. cortical bone), skeletal element, sample location within that element (e.g. epiphysis, metaphysis, and diaphysis) and the age of the individual [10, 14, 21–24]. Thus, a multi-tissue approach can assist in reconstructing life histories from birth to the week prior to death. For a successful application of isotope research in forensic casework, the preservation of the antemortem isotope signatures in different tissues of the body after death is of paramount importance. However, the isotope composition of human tissues is potentially compromised by diagenetic processes: chemical weathering that changes the chemical and physical characteristics and nature of the tissues through the exchange of components between the tissue and the (burial) environment [25].

Archaeological bone and dentine apatite are rarely targeted due to their known susceptibility to diagenetic alterations. These tissues, as well as organic human remains such as hair and nail keratin, which rarely survive in the archaeological record, are more commonly used in medico-legal investigations. However, there is very little information available from forensic and experimental research on the effects of diagenesis on the biogenic isotopic signatures of these body tissues [26–28].

This small-scale study evaluates the recoverability of antemortem Sr–Pb isotope ratios from buried human tissues frequently consulted in forensic investigations, including scalp hair, bone (pelvic iliac bone and tibia), and tooth enamel and dentine. Body donations were placed in three different burial contexts at the Forensic Anthropology Research Facility (FARF), managed by the Forensic Anthropology Centre at Texas State University (FACTS), San Marcos, TX. The preplacement and post-recovery isotope compositions of the different tissue types were compared to gain insights into their suitability for identification of geographical origins and in reconstruction of human mobility patterns in forensic investigations.

## Material and methods

### Taphonomic experiment

The body donations of five females, aged between 61 and 91 years, were placed unclothed to decompose outdoors at FARF between April 2015 and May 2021. Biological information on the donors and information on the tissue samples are provided in Table 1. The time of death ranged between 2 and 10 days prior to placement. Bodies were stored in coolers at 4 °C prior to placement. Each body was in a fresh stage of decomposition upon placement, based on the decomposition scoring method described in [29].

**Table 1:** Donation and sample data. All willed-donated bodies were biological females, aged between 61 and 91 years at death. Key: A = intake sample; B = sample taken during active decomposition; C = sample taken during retrieval of the remains in skeletonised stage at latest; * = day before interment/placement; ** = day of interment/placement

Donation	FARF code	Time as resident of Texas immediately prior to death	Time since death upon placement in days	Deposition context	Sample ID	Collection date (dd-mm-yyyy)	Time since start experiment in days
1	D18-2015	~ 10 years	10	Open pit	A	28–04-2015	− 1*
					B	04–05-2015	6
					C	03–12-2015	219
2	D22-2015	7 months	2	Burial	A	07–05-2015	0**
					C	17–08-2017	814
3	D33-2015	Entire life	3	Burial	A	24–06-2015	0**
					C (hair)	21–08-2017	790
					C (tooth)	24–08-2017	793
					C (bone)	09–03-2018	990
4	D57-2015	Entire life	10	Open pit	A	19–10-2015	− 1*
					B	26–10-2015	7
					C (hair)	15–11-2015	28
					C (rest)	09–03-2018	872
5	D43-2017	23 years	9	Mummification	A	03–08-2017	− 1*
					B-1	25–08-2017	21
					B-2	05–03-2018	215
				Burial	C (tooth)	20–05-2021	1022
					C (rest)	23–05-2021	1025

Body donations 1 and 4 were placed in hand-dug oval-shaped pits in a flexed position (depicted in Fig. 4 in [30]), measuring approximately 65 cm in width, 95 cm in length, and 70 cm in depth. These pits remained open throughout the study and were observed directly while decomposition of soft tissue and skeletonisation occurred. During the experiment, two extreme precipitation events occurred (flash floods), flooding the open pits in which body donations 1 and 4 were placed. Water subsided slowly due to the clay-rich soils, partially submerging donation 1 for a total of 15 days (from day 17 since the start of the experiment) and donation 4 for three days (from day 11 since the start of the experiment). Body donations 2 and 3 were buried in a flexed position in shallow oval-shaped pits using the same soil to backfill the pits, simulating shallow clandestine burial contexts. Donation 5 was allowed to desiccate for 7 months through solar and aeolian desiccation in a flexed body position. After that time, the mummified body was buried in an oval pit, similarly to donations 2 and 3, and excavated in May 2021.

### Sample collection

Two bone samples, one single-rooted tooth, and one scalp hair sample were collected from each of the five body donors prior to their placement to decompose outdoors (“A”), during active decomposition (“B”, hair sampled from donations placed in open pits only) and after retrieval of the skeletonised remains (“C”, Table 1). In addition, soil samples from the burial environment and elsewhere on the FARF terrain were taken for reference purposes (“S”).

#### Soil

Soil samples from the burial location were collected prior to placement of the body donations outdoors at a depth of 30–40 cm (“SA” samples) and again after decomposition (“SC” samples), but at different depths varying between 20 cm (above abdomen) and 70 cm (see Table 2). Circa 100 mL of soil was collected with a trowel from the fill of the pits during their excavation. In addition, a control soil sample was collected with a trowel from an unused part of FARF, 50 m away from the experiment area and approximately 20 cm below the surface (sample S0).

**Table 2:** Sr isotope data from the soil samples. Key: S = soil; A = intake sample; C = sample taken during retrieval of the remains in skeletonised stage at latest, after decomposition; [Sr]: concentration Sr in ppm in leach; [Pb] = concentration Pb in ppb in leach; * = most samples from donation 3 included in the present study were collected in August 2017, along with soil sample S3C-1. A hurricane delayed the recovery of other skeletal remains (bones), which took place in March 2018 when S3C-2 was collected. All two standard errors (2SE) of ^87^Sr/^86^Sr are < 0.00001

Donation	VU code	Decomposition context	Description	Time since start experiment in days	^87^Sr/^86^Sr	[Sr]	[Pb]
-	S0		Random soil sample from unused part of site	0	0.709086	1.48	1.29
1	S1A	Open pit	Initial soil sample prior to body placement (30–40 cm deep)	0	0.709182	2.33	10.57
	S1C		Sample upon retrieval of the remains	219	-	-	-
2	S2A	Burial	Initial soil sample prior to body placement (30–40 cm deep)	0	0.709389	1.63	30.32
	S2C-1		Sample upon retrieval of the remains (20 cm deep, above abdomen)	814	0.709716	1.74	7.26
	S2C-2		Second sample upon retrieval of remains (40 cm deep, in abdomen)	814	0.709671	1.89	46.42
3	S3A	Burial	Initial soil sample prior to body placement (30–40 cm deep)	0	0.709441	1.26	7.20
	S3C-1		Sample upon retrieval of the remains (2017, 60–70 cm deep, feet area)	793	0.709911	1.26	23.93
	S3C-2*		Sample upon retrieval of the remains (2018, 60–70 cm deep, feet area)	990	0.709822	1.51	10.06
4	S4A	Open pit	Initial soil sample prior to body placement (30–40 cm deep)	0	0.709210	2.38	7.37
	S4C		Sample upon retrieval of the remains (30–40 cm deep)	872	0.709451	1.77	19.29
5	S5A	Mummification/ burial	Initial soil sample prior to body placement (30–40 cm deep)	0	0.709417	1.51	0.60
	S5C		Sample upon retrieval of the remains (25 cm deep, in abdomen)	1025	0.709463	-	-

#### Scalp hair

The scalp hair samples collection and analysis of donations 1 to 4 has been the subject of an earlier study [28], except for the Pb isotope data of the H1B, H3C, and H4B samples that are included in the present study, together with donation 5. Scalp hair “A” samples of the five fresh body donations included in the present study were collected during the intake of the donation. During each sample collection campaign, circa 100 hair strands were collected using tweezers and extracted at the root when possible. The “B” samples from open pit donations (1 and 4) were collected approximately one week after placement when the body was in a state of active decomposition. The “B” samples of donation 5 were collected during the process of mummification in August 2017 and again in March 2018, respectively 23 and 215 days after the start of the experiment. In March 2018, the mummified body was buried for a further 3 years and 2 months, until May 2021. All “C” samples were taken during retrieval of the remains in skeletonised state.

#### Teeth

A lower central incisor was extracted upon intake (“A” samples: World Dental Federation notation (hereafter: FDI) 31 or 41) for all donations, except for donation 1, from which a lower lateral incisor (FDI 32) was collected. Another single-rooted tooth (FDI 31 or 41; left canine 43 in donation 1) was extracted after decomposition at the end of the experiment (“C” samples), except for donation 5 for which dentine was not available for analysis. Extraction was performed using two dental elevators to minimise tooth breakage.

#### Bone

During intake of the fresh body donations, approx. 1 cm^3^ pre-burial samples (“A”) of the iliac crest of the right ilium and the right anterior mid-shaft of the tibia were collected from each donor using a disposable scalpel and a 12 V Dremel cordless lithium-ion drill with a circular diamond wheel drill bit. After decomposition outdoors, 1 cm^3^ bone samples of the iliac crest of the left ilium and the left anterior mid-shaft of the tibia were collected from each donor to ensure similar distribution of compact and cancellous bone in both the “A” and “C” samples.

### Sample storage

Bone, tooth, and soil samples were packed into individual Ziploc storage bags, which were placed inside another individual Ziploc bag. Hair strands were aligned along the direction of growth and subsequently wrapped in aluminium foil and packaged in two Ziploc storage bags. Samples were transferred to a lockable freezer and stored at − 20 °C until shipped overnight on dry ice to the Vrije Universiteit Amsterdam, the Netherlands. Upon arrival, the samples were immediately documented and transferred to a lockable freezer at − 20 °C. A detailed description of sample preparation is given in Supplementary Information 1.

## Analytical details

### Ion-exchange chemistry

Disposable polyethylene pipette-tip columns fitted with a polyethylene filter (mesh 35 μm) were used for the purification of Pb. The columns were loaded with 200 μL of Bio-Rad® AG 1-X8 200–400 mesh resin. A detailed description of the protocol can be found in Klaver et al. [31]. In short, the matrix (pre-fraction) was collected in acid-cleaned Teflon beakers, dried, and then dissolved in 500 μL of 3 M HNO_3_ for Sr purification. The purified Pb fractions were collected, and sub-samples were transferred to pre-cleaned MC-ICP-MS vials to create 1% HNO_3_ solutions containing 50 ppb, 20 ppb, 10 ppb, or 5 ppb of Pb, depending on the sample size.

For Sr purification, similar pipette-tip columns were used and loaded with 80 μL of Sr selective extraction chromatographic resin from Eichrom Technologies and cleaned three times with alternating 3 M HNO_3_ and Milli-Q before conditioning with 500 μL of 3 M HNO_3_. The sample was loaded, and the matrix was washed out with two rinses of 900 μL of 3 M HNO_3_. The Sr fraction was eluted with 800 μL of Milli-Q and collected in acid-pre-cleaned Teflon beakers. The purified samples were dried, then nitrated overnight to remove organic material, and subsequently dried and transferred to the mass spectrometer laboratory.

### Mass spectrometry

Strontium samples were redissolved in 2 μL of 10% HNO_3_ and 50% loaded with 2 μL of TaCl_5_ on cleaned and outgassed single annealed rhenium filaments. The Sr isotope compositions were measured using a Thermo Scientific™ Triton Plus™ thermal ionisation mass spectrometer (TIMS). The Sr isotope ratios were determined using a static routine and corrected for mass fractionation to a ^86^Sr/^88^Sr ratio of 0.1194 [32]. NIST® SRM® 987 gave an average of 0.710252 ± 0.000012 (2 s) during the course of this study (*N* = 37). Over the period 2017–2021, the method yielded 0.710254 ± 0.000018 (2 s; *N* = 433). All measurements were normalised to an accepted value of 0.710240 for NIST® SRM® 987. The ^87^Sr/^86^Sr are reported plus minus two standard errors (2SE), representing the typical measurement precision obtained from 240 cycles of 8.1 s integration time (12 blocks of 20 cycles) within each run. The total procedural blanks (*N* = 12) contained less than 45 pg strontium.

In contrast to the earlier published hair samples, which were analysed using the double spike method [31] on the TIMS equipped with 10^13^Ω amplifiers at the Vrije Universiteit Amsterdam [28], all new Pb isotope analyses presented in this paper were performed on a Neptune MC-ICP-MS equipped with 10^12^Ω amplifiers at the Institut für Mineralogie, Westfälische Wilhelms-Universität Münster in Germany. During the analytical run, repeated measurements of the Münster in-house standard yielded 16.9926 ± 0.0014 for ^206^Pb/^204^Pb, 15.5102 ± 0.0018 for ^207^Pb/^204^Pb, and 36.7546 ± 0.0055 for ^208^Pb/^204^Pb (2 s; *N* = 19), which is consistent with the long-term precision (2015–2021) of the Neptune. The total procedural and column blanks (*N* = 12) contained on average 22 pg lead.

## Results

### Soil samples

**Fig. 1:**
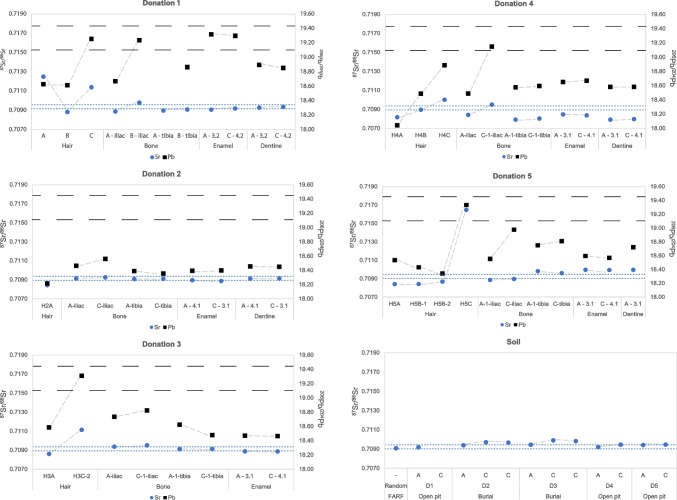
^87^Sr/^86^Sr (blue circle) and ^206^Pb/^204^Pb (black square) per donation (D1–5), and soil ^87^Sr/^86^Sr data. Error bars are smaller than the symbols. Key: –– –– –– = local ^206^Pb/^204^Pb signature (black); - - - = local ^87^Sr/^86^Sr signature (blue). Local soil ^87^Sr/.^86^Sr from [26]

The soil Sr isotope data are presented in Table 2 and Fig. 1. Post-decomposition sample S1C from donation 1 was not received in Amsterdam. All analysed samples show a consistent trend of increased ^87^Sr/^86^Sr in the post-decomposition “C” samples (average ^87^Sr/^86^Sr_A_ = 0.70933, average ^87^Sr/^86^Sr_C_ = 0.70976, and average ^87^Sr/^86^Sr_A–C_ = 0.00043). In contrast to the other burials, the isotopic difference between the “A” and “C” soil samples in donation 5 was limited (^87^Sr/^86^Sr_A–C_ = 0.000046). All intake and post-decomposition samples exhibit higher ^87^Sr/^86^Sr than the FARF soil sample (S0) collected from an unused part of the site (^87^Sr/^86^Sr = 0.70909).

The variation in Sr concentration [Sr] in the soil samples is limited (1.26–2.38 ppm). The [Sr] in the “C” samples varies compared to the “A” samples, with both minor increases and decreases in [Sr] observed (e.g. donations 2 and 4, respectively, Table 2). The Pb concentration data also show no consistent variation. In donations 3 and 4, a significant increase in [Pb] is seen between the “A” and “C” samples, with a subsequent time-dependent decrease in donation 3 (S3C-2). In contrast, the soil [Pb] in the “C” in donation 3 is slightly higher in the deeper sampled “C-2” sample (+ 6.1 ppb), but lower in the shallower collected “C-1” sample (– 23.06 ppb) compared to the “A” sample (Table 2).

### Human tissue samples

**Fig. 2:**
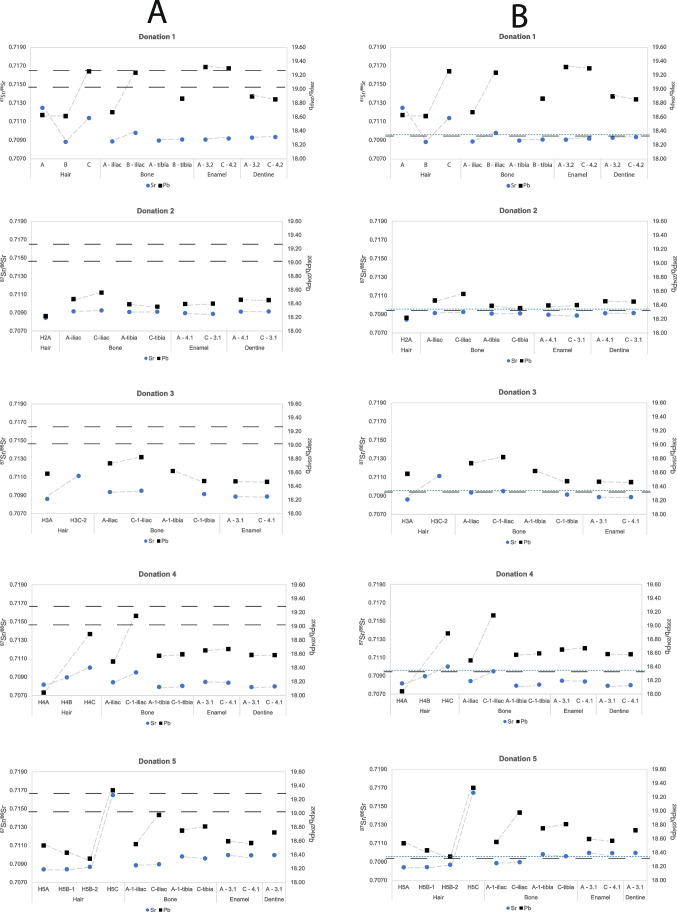
**A** ^207^Pb/^204^Pb and ^208^Pb/^204^Pb ratios. Key: –– –– –– = local ^208^Pb/^204^Pb signature (black). **B** ^207^Pb/^206^Pb and ^208^Pb/^206^Pb ratios of hair, bone, enamel, and dentine samples from donations 1–5. Key: –– –– –– = maximum local ^208^Pb/^206^Pb signature (black); - - - = maximum local ^207^Pb/^206^Pb signature (blue). Error bars are smaller than the symbols

The Sr and Pb isotope data are presented in Table 3 and Figs. 1 and 2.

**Table 3:** Sr–Pb isotope data from samples from donations 1 to 5. Key: H: hair samples; B: bone samples; E: enamel samples; D: dentine samples; A: intake sample; B: sample taken during active decomposition; C: sample taken during retrieval of the remains in skeletonised stage at latest; [Sr] and [Pb]: concentration Sr and Pb in ppm; -: no data, analysis failed; *: inaccurate data, < 60 cycles/measurements. Dentition codes of E and D samples follow those of the FDI World Dental Federation. References refer to: 1: Kootker et al. 2020 [28]; 2: Pb isotope data published in the present study, Sr in Kootker et al. 2020 [28]; 3: this study. ^206^Pb/^207^Pb, ^208^Pb/^207^Pb, and all standard errors (2SE) are provided in Supplementary Information 2–SI1

Donation	VU code	[Sr]	[Pb]	^87^Sr^/86^Sr	^206^Pb/^204^Pb	^207^Pb/^204^Pb	^208^Pb/^204^Pb	^207^Pb/^206^Pb	^208^Pb/^206^Pb	Reference
1	H1A	-	-	0.71252	18.631	15.616	38.285	0.8382	2.0550	1
	H1B	-	-	0.70885	18.616	15.638	38.326	0.8400	2.0588	2
	H1C	-	-	0.71142	19.258	15.683	38.799	0.8144	2.0147	1
	B1A-iliac	60.4	1.2	0.70890	18.671	15.646	38.382	0.8380	2.0557	3
	B1C-iliac	58.6	5.7	0.70981	19.238	15.682	38.850	0.8151	2.0194	3
	B1A-tibia	70.8	0.9	0.70899	-	-	-	-	-	3
	B1C-tibia	72.8	1.1	0.70910	18.867	15.644	38.492	0.8291	2.0402	3
	E1A-32	113.1	10.1	0.70909	19.320	15.698	38.893	0.8125	2.0131	3
	E1C-43	83.9	5.2	0.70921	19.301	15.688	38.856	0.8128	2.0132	3
	D1A-32	59.5	17.8	0.70929	18.897	15.651	38.526	0.8282	2.0387	3
	D1C-43	56.5	13.4	0.70937	18.856	15.647	38.497	0.8298	2.0417	3
2	H2A	-	-	0.70845	18.218	15.672	38.167	0.8603	2.0951	1
	H2C	-	-	-	-	-	-	-	-	-
	B2A-iliac	80.6	0.5	0.70914	18.465	15.625	38.176	0.8462	2.0675	3
	B2C-iliac	74.5	0.5	0.70926	18.561	15.620	38.210	0.8416	2.0586	3
	B2A-tibia	70.7	0.9	0.70908	18.389	15.611	38.094	0.8489	2.0715	3
	B2C-tibia	72.8	1.1	0.70911	18.355	15.606	38.092	0.8502	2.0753	3
	E2A-41	126.4	2.8	0.70896	18.395	15.617	38.191	0.8490	2.0761	3
	E2C-31	133.6	3.1	0.70888	18.399	15.620	38.199	0.8490	2.0762	3
	D2A-41	52.8	3.5	0.70913	18.456	15.624	38.189	0.8466	2.0692	3
	D2C-31	66.7	3.4	0.70914	18.450	15.622	38.184	0.8467	2.0696	3
3	H3A	-	-	0.70861	18.584	15.635	38.373	0.8413	2.0648	1
	H3C-2	-	-	0.71114	19.311	15.586	38.925	0.8120	2.0160	2
	B3A-iliac	66.2	0.6	0.70937	18.736	15.660	38.415	0.8358	2.0504	3
	B3C-1-iliac	75.1	0.3	0.70952	18.824	15.662	38.487	0.8320	2.0446	3
	B3A-1-tibia	81.1	0.8	0.70913	18.624	15.647	38.326	0.8402	2.0579	3
	B3C-1-tibia	92.4	1.1	0.70914	18.478	15.629	38.213	0.8458	2.0681	3
	E3A-31	207.8	5.6	0.70888	18.471	15.628	38.200	0.8461	2.0681	3
	E3C-41	205.9	4.8	0.70887	18.465	15.619	38.172	0.8459	2.0673	3
4	H4A	-	-	0.70818	18.042	15.504	37.843	0.8593	2.0975	1
	H4B	-	-	0.70896	18.487	15.626	38.237	0.8452	2.0682	2
	H4C	-	-	0.71002	18.888	15.657	38.521	0.8290	2.0395	1
	B4A-iliac	47.4	0.6	0.70843	18.491	15.627	38.253	0.8451	2.0687	3
	B4C-1-iliac	90.3	6.3	0.70950	19.150	15.683	38.754	0.8189	2.0237	3
	B4A-1-tibia	391.4	1.9	0.70792	18.575	15.612	38.263	0.8405	2.0599	3
	B4C-1-tibia	284.1	1.5	0.70804	18.595	15.635	38.335	0.8408	2.0616	3
	E4A-31	55.3	5.1	0.70847	18.651	15.634	38.430	0.8382	2.0605	3
	E4C-41	66.9	6.1	0.70838	18.670	15.632	38.428	0.8373	2.0583	3
	D4A-31	404.8	4.7	0.70792	18.584	15.635	38.332	0.8413	2.0626	3
	D4C-41	482.8	7.3	0.70798	18.583	15.648	38.362	0.8420	2.0644	3
5	H5A	-	-	0.70842	18.535	15.654	38.346	0.8445	2.0688	3
	H5B-1	-	-	0.70843*	18.433	15.633	38.253	0.8481	2.0752	3
	H5B-2	-	-	0.70869	18.344	15.645	38.241	0.8529	2.0846	3
	H5C	2.9	3.3	0.71648	19.334	15.686	38.892	0.8113	2.0115	3
	B5A-1-iliac	59.7	0.2	0.70886	18.554	15.644	38.266	0.8432	2.0624	3
	B5C-iliac	72.5	0.3	0.70897	18.977	15.672	38.570	0.8258	2.0325	3
	B5A-1-tibia	71.2	0.6	0.70983	18.751	15.653	38.373	0.8348	2.0464	3
	B5C-tibia	67.4	0.5	0.70961	18.810	15.655	38.403	0.8323	2.0417	3
	E5A-31	96.4	1.8	0.70997	18.596	15.636	38.272	0.8408	2.0581	3
	E5C-41	102.1	2.9	0.70994	18.571	15.639	38.272	0.8421	2.0609	3
	D5A-31	78.5	1.8	0.70997	18.722	15.649	38.354	0.8358	2.0485	3
	D5C-41	-	-	-	-	-	-	-	-	-

#### Hair keratin

The most striking differences in Sr isotope ratios are seen in the hair keratin data. The intra-individual isotopic shift between the intake and recovery samples exceeds 0.001 in donations 1 to 4 [28]. The difference in ^87^Sr/^86^Sr between the “A” and “B” samples in donation 5 (collected during fresh and mummification stages, respectively) is limited (^87^Sr/^86^Sr_A–B_ =  < 0.0003). However, after burial of donation 5 for 807 days (Table 1), the strontium isotope composition of the “C” samples shifted to 0.71648, an increase of 0.0078 and in strong contrast with the average of donation 5’s “B” samples (0.70856).

The hair Pb isotope data have been discussed earlier for donations 1 to 4 [28]. In short, biogenic Pb isotope ratios were altered within days of burial. In donation 5, the difference in Pb isotope composition between the “A” (intake) and the “B” (mummified) samples is relatively limited (Table 3), but outside analytical error (Supplementary Information 2—SI1).

#### Bone

The ^87^Sr/^86^Sr of the “A” (intake) and “C” (sample taken during retrieval of the remains) iliac bone samples from donations 1 and 4 (open pit burial) show significant changes from 0.70890 and 0.70843 to 0.70981 and 0.70950 (intra-individual ^87^Sr/^86^Sr_A–C_ = 0.009 and 0.011, respectively). In contrast, a smaller increase to higher ^87^Sr/^86^Sr is observed in the post-burial iliac bone samples from donations 2, 3, and 5 (buried and mummified and subsequently buried; all ^87^Sr/^86^Sr_A–C_ = 0.0001). Similarly, the ^87^Sr/^86^Sr of the post-burial tibia bone samples from donations 2 and 3 showed little alteration compared with the pre-burial samples (^87^Sr/^86^Sr_A–C_ ≤ 0.00003). More pronounced shifts are observed in the pre- and post-burial tibia samples of the open pit donations 1 and 4 (both ^87^Sr/^86^Sr_A–C_ = 0.0001), although this difference is less marked than in the iliac samples (^87^Sr/^86^Sr_A–C_ = 0.001). The isotopic shift between the “A” and “C” samples in the tibia bone from donation 5 is more pronounced, i.e. ^87^Sr/^86^Sr_A–C_ = 0.0002, and in the opposite sense compared to all other samples, i.e. towards a less radiogenic ratio (0.70983 to 0.70961).

Isotopic differences in Pb varying between < 0.001(^207^Pb/^206^Pb, donation 4) and 0.072 (^208^Pb/^204^Pb, donation 4) are observed between the “A” and “C” cortical bone samples (tibia), with the exception of donation 3. In this donation, all Pb isotope data of the tibia “C” samples show a more pronounced shift than observed in the trabecular (iliac) samples (up to 0.146 for the ^206^Pb/^204^Pb, with 0.09 for the same ratio in the iliac sample, Table 3). In all other donations, the isotopic shift between the “A” and “C” samples is significantly larger for the trabecular bone samples. The differences vary between 0.002 (^207^Pb/^204^Pb, donation 3) and 0.66 (^206^Pb/^204^Pb, donation 4).

#### Dental elements

Variations in the Sr isotope compositions of the enamel are smaller than 0.00009, except for donation 1 (^87^Sr/^86^Sr_A–C_ = 0.0001). The intra-individual ^87^Sr/^86^Sr_A–C_ differences between “A” and “C” dentine samples are also small (^87^Sr/^86^Sr_A–C_ =  < 0.00008). The lead isotope compositions of the enamel and dentine samples are consistent (^20x^Pb/^20x^Pb_A–C_ =  ≤ 0.002), apart from ^206/207/208^Pb/^204^Pb that exhibit larger shifts (up to 0.04), see Table 4). In contrast to the ^87^Sr/^86^Sr in donation 1, the ^206, 207, 208^Pb/^204^Pb and ^207,208^Pb/^206^Pb data of the lateral incisor (“A”) and canine (“C”) are comparable (difference A–C = 0.042, Table 3), although the differences are larger than the analytical error (2SE =  ≤ 0.003. See Supplementary Information 2—SI1).

**Table 4:** Differences in Sr-Pb isotope composition of hair keratin, dental elements and bone tissues between the pre-burial “A” samples and the post-excavation “C” samples per donation (D)

	^**87**^**Sr/**^**86**^**Sr**								^**208**^**Pb/**^**204**^**Pb**		
	A–C						A–C				
	D1	D2	D3	D4	D5		D1	D2	D3	D4	D5
Hair	0.0011		− 0.0025	− 0.0018	− 0.0081	Hair	− 0.513	-	− 0.552	− 0.678	− 0.546
Iliac	− 0.0009	− 0.0001	− 0.0001	− 0.0011	− 0.0001	Iliac	− 0.468	− 0.034	− 0.072	− 0.502	− 0.304
Tibia	0.0000	− 0.0001	0.0000	− 0.0001	0.0002	Tibia	-	0.001	0.113	− 0.072	− 0.030
Enamel	− 0.0001	0.0000	0.0000	0.0001	0.0000	Enamel	0.036	− 0.008	0.028	0.002	0.000
Dentine	− 0.0001	0.0001		− 0.0001		Dentine	0.029	0.005	-	− 0.030	-
	^**206**^**Pb/**^**204**^**Pb**								^**207**^**Pb/**^**206P**^**b**		
	A–C						A–C				
	D1	D2	D3	D4	D5		D1	D2	D3	D4	D5
Hair	− 0.627	-	− 0.727	− 0.846	− 0.799	Hair	0.024	-	0.029	0.030	0.033
Iliac	− 0.567	− 0.095	− 0.088	− 0.659	− 0.423	Iliac	0.023	0.005	0.004	0.026	0.017
Tibia	-	0.034	0.146	− 0.020	− 0.059	Tibia	-	− 0.001	− 0.006	0.000	0.003
Enamel	0.019	− 0.003	0.006	− 0.018	0.025	Enamel	0.000	0.000	0.000	0.001	− 0.001
Dentine	0.042	0.006	-	0.001		Dentine	− 0.002	0.000	-	− 0.001	-
	^**207**^**Pb/**^**204**^**Pb**								^**208**^**Pb/**^**206**^**Pb**		
	A–C						A–C				
	D1	D2	D3	D4	D5		D1	D2	D3	D4	D5
Hair	− 0.067	-	0.049	− 0.153	− 0.033	Hair	0.040	-	0.049	0.058	0.057
Iliac	− 0.036	0.005	− 0.002	− 0.057	− 0.028	Iliac	0.036	0.009	0.006	0.045	0.030
Tibia	-	0.005	0.018	− 0.023	− 0.001	Tibia	-	− 0.004	− 0.010	− 0.002	0.005
Enamel	0.010	− 0.003	0.009	0.002	− 0.003	Enamel	0.000	0.000	0.001	0.002	− 0.003
Dentine	0.004	0.002	-	− 0.012	-	Dentine	− 0.003	0.000	-	− 0.002	-

## Discussion

### Soil data

The strontium isotope composition of the S0, SA and SC soil leachate samples collected at each burial location is comparable to the surface and open grassland soil samples taken at FARF (~ 0.7088–0.7096, mean ± 2 s). The range extends to 0.7098 if the soil samples taken from the forested part of FARF are included (Table 21 in [26]). In the present study, the soil’s lead isotope composition was not analysed. FARF soil reference data, however, are available ([26], mean ± 2 s): ^206^Pb/^204^Pb = 19.283 ± 0.156, ^207^Pb/^204^Pb = 15.765 ± 0.297, ^208^Pb/^204^Pb = 38.685 ± 0.105, ^208^Pb/^206^Pb = 2.005 ± 0.011, and ^207^Pb/^206^Pb = 0.812 ± 0.005.

The consistent trend of increased ^87^Sr/^86^Sr in the post-placement compared to the preplacement samples in all but donation 5 suggests that active decomposition, i.e. liquefaction and the release of decomposition fluids into the soil, may have impacted the soil ^87^Sr/^86^Sr. Donation 5 was placed in the grave desiccated and did not release decomposition fluids into the soil, potentially resulting in a less chemical reaction in the soil. However, the observed increase in post-burial ^87^Sr/^86^Sr compared to the pre-burial ^87^Sr/^86^Sr (Table 4), is not easily explained solely by the decomposition of the bodies, as there is significantly less Sr in the body (blood serum, [Sr] ca. 20–200 ppb [33, 34]) than present in the FARF soil leachates ([Sr] 1–3 ppm in the present study, although lower concentrations (170–300 ppb) are reported in [26]). Due to the low Sr concentration in precipitation in Texas ([Sr] 2–8 ppb, [26]), rainwater cannot have contributed significantly to the increase in soil ^87^Sr/^86^Sr. The Sr concentration is considerably higher in the Texas groundwater ([Sr] 8–10 ppm, [26]), but since all donations were buried above the groundwater level, the effect of groundwater on post-decomposition soil ^87^Sr/^86^Sr is also considered limited. Nevertheless, the influence of the hydrology of the site during the burial period cannot be overlooked. During the experiment, two extreme precipitation events (flooding) occurred, filling both open pit burials (donations 1 and 4) with water for 15 and 3 days, respectively. These events potentially influenced the water balance of the soil and its Sr isotope composition.

Notably, the ^87^Sr/^86^Sr of the post-burial samples from donation 3 seem to indicate that soil Sr isotope compositions are likely to return to their original ratios. The Sr ratio rose to 0.709911 (S3C-1) after the active decay of the body, from an initial ratio of 0.709441 (S3A). Eight months after the recovery of the body, the Sr ratio had decreased to 0.709822, a difference of 0.000099. The effect of sub-sampling, however, needs to be fully evaluated before these preliminary data can be ascribed to the influence of body decomposition. Soils from a single substrate can exhibit wide ranges of ^87^Sr/^86^Sr due to the variation in individual mineral content of the different samples (e.g. [35]). The differences in ^87^Sr/^86^Sr between the collected soil samples could therefore be partly explained by the varying mineral content of the samples, whether induced by decomposition fluids or precipitation (i.e. the breakdown of minerals or ion exchange with minerals).

Soil chemistry, acidity, and hydrology were not analysed (yet) or monitored between placement and recovery of the body donations. Additional research into the local soil hydrological processes operating at FARF during actualistic experiments, as well as into soil lithology and chemical properties, is needed to better understand the effect of decomposition, precipitation, and groundwater on the soil’s ^87^Sr/^86^Sr.

### Human data

Before discussing the isotopic shifts observed per tissue type, it is crucial to discuss two topics: (1) the human biogenic (pre-burial) Sr and Pb isotope compositions in relation to the soil Sr–Pb isotope data, and (2) the natural within-tissue variation in Sr–Pb isotope composition.

#### Human tissue-soil variations

The pre-burial soil Sr isotope data generated in the present study varies between ca. 0.7090 and 0.7094. This range corresponds to the pre-burial “A” data of the bone and dental elements of donations 1, 2, and 3, and the iliac sample in donation 5. Pre-burial bone and dental isotope compositions of donations 4 and 5 (except the iliac sample) differ from the baseline soil data (> 0.0002). Consequently, if the soil Sr isotope composition was of significant influence on the diagenetic alteration of the human tissue ^87^Sr/^86^Sr, minor shifts in ^87^Sr/^86^Sr between the “A” and “C” burials of donations 1, 2, and 3, and in the iliac sample of donation 5 would be expected.

The pre-burial hair samples all exhibit significantly different ^87^Sr/^86^Sr to the soil (donation 1 higher (0.7125) and donations 2, 3, 4, and 5 lower (0.7081–0.7086)). Previous research conducted on these samples, however, concluded that sample pretreatment may affect the measured ^87^Sr/^86^Sr; in this case, it may have insufficiently removed exogeneous Sr isotope signatures [28]. This present study applied the same protocol for donation 5 to allow inter-study comparisons of ^87^Sr/^86^Sr.

The soil’s ^206,208^Pb/^204^Pb and ^207,208^Pb/^206^Pb given in Sect. 6.1 differ from nearly all pre-burial “A” samples, except for the enamel of tooth 3.2 from donation 1, which is comparable. In contrast, consistent with the lower overall terrestrial variation in ^207^Pb/^204^Pb, all pre-burial ratios are comparable with that of the soil.

#### Intra-tissue variation

Another potentially significant variable is the naturally occurring variation in Sr–Pb isotopic composition within human tissues. For the hair samples bulk, samples were collected, averaging out the variations in Sr–Pb isotope composition that occur between individual hair strands because they start and stop growing at different times. Nevertheless, different bulk samples of the same individual may exhibit different Sr–Pb isotope compositions. Naturally occurring intra-individual differences in hair keratin Sr–Pb isotope composition have not been fully quantified, although a study by Font et al. [36] observed differences up to ca. 0.0004 in ^87^Sr/^86^Sr and 0.008 in ^206^Pb/^207^Pb for a female individual who resided in Amsterdam, the Netherlands, which is ca. 425 times the analytical error in the present study. Consequently, significant intra-individual variations in hair keratin Sr–Pb isotope composition are likely.

Although research has been conducted on intra-bone variation in stable isotope composition of bone [37, 38], little is known about possible intra-bone and intra-skeletal isotopic variations in Sr–Pb. Bone is composed of a mixture of trabecular and cortical bone, and full-grown long bones can be divided into separate regions: the diaphysis, or shaft, and the metaphysis. The remodelling rate is fastest in the trabecular bone and the metaphysis, potentially resulting in isotopic differences between the different types of bone and locations within the bone. To date, intra-bone isotopic variations in Sr–Pb have not been quantified, but they are likely to exceed those in enamel (see below) due to continuous remodelling.

Intra-enamel variation in enamel ^87^Sr/^86^Sr in the modern Dutch population is 0.0002 [39]. Since comparative data are not available from American populations, differences in ^87^Sr/^86^Sr between pre- and post-burial samples of less than 0.0002 are therefore difficult to interpret. To date, intra-dental elemental variations in Pb isotope composition have not been quantified. In addition, intra-dental elemental variations in Sr–Pb isotope composition of dentine have not been examined. However, based on the extended time of enamel mineralisation and maturation, primary dentine formation (i.e. dentinogenesis lasts for more than 3 years after crown completion [16]), and the deposition of secondary and tertiary dentine during life [16], the Pb isotope composition of enamel and the Sr–Pb isotope composition of dentine samples are expected to vary among individuals who have moved and or changed diet.

### Tissue Sr-Pb

#### Hair keratin

The hair samples of four of the five body donors (1–4) were the subject of an earlier publication, which found that chemical and microbiological degradation of hair fibres occurred rapidly after placement of the body outdoors. Scalp hair H–Pb–Sr isotope ratios were altered within days of placement of the body outdoors [28]. The results from donation 5 add to our knowledge about the use of keratin in mummified forensic and archaeological contexts. A small and insignificant (see Sect. 6.2.2) isotopic shift in Sr between intake “A” and “B-2” samples was observed (^87^Sr/^86^Sr_A–B_ =  < 0.0003). Furthermore, the Pb isotope data vary little; ≤ 0.015 for all ratios, except for ^206^Pb/^204^Pb (0.2) and ^208^Pb/^204^Pb (0.1). The latter two ratios exhibit the highest analytical error (0.01 and 0.02, respectively), indicating less precise but reliable data.

The isotopic difference in Sr between the “H5B-1” and “H5B-2” samples (^87^Sr/^86^Sr_B1-B2_ =  < 0.00027), corresponding to different states of mummification (collected at day 21 and 215, respectively, see Table 2), is also below the 0.0004 intra-individual difference observed in [36]. The variations in Pb isotope composition are also ≤ 0.01 for all ratios except for ^206^Pb/^204^Pb (0.09), thus significantly exceeding the analytical uncertainties (Supplementary Information 2—SI1). Nevertheless, the possible effect of prolonged exposure to rainwater, dust, and UV light cannot be excluded. As hair keratin samples are likely to contain low concentrations of strontium (ca. 0.1–30 ppm, depending on hair colour, see, e.g. [40, 41]) compared to, for instance, soil, precipitation, and dust, relatively little material is needed to interact with the hair to cause an isotopic shift.

The significant effect of burial on the hair Sr–Pb isotope composition can be seen in the “H5C” sample, collected 810 days after burial. The hair’s ^87^Sr/^86^Sr shifted significantly (0.7086 to 0.7164, Table 3, Fig. 2), and the ^206^Pb/^204^Pb and ^207,208^Pb/^206^Pb shifted to local soil Pb isotope composition [26] (Supplementary Information 2—SI2). The ^208^Pb/^204^Pb of the “H5C” sample shifted from 38.35, lower than the expected soil’s ^208^Pb/^204^Pb, to 38.892, which is slightly higher (0.10) than the local range. Given the clear shift to local soil Pb isotope compositions in the other ratios, it is likely that a ^208^Pb/^204^Pb of 38.892 also reflects a diagenetic, local, and isotopic ratio.

Although prolonged exposure to soil may be an obvious explanation for the changes seen in the Sr–Pb isotope composition, particularly for the Pb isotope data, the fact that the ^87^Sr/^86^Sr shift is so far above the ^87^Sr/^86^Sr of soil shows that more factors are at play than interaction with bulk soil. More research is needed to better understand the mechanisms involved. Additional research into cleaning methodologies that remove diagenetic isotope signatures/contaminations from hair keratin samples without the removal of all biogenic Sr–Pb, is forthcoming [42].

#### Bone

The difference between pre- and post-placement ^87^Sr/^86^Sr in the iliac bone (^87^Sr/^86^Sr_A–C_ = 0.00047) is on average five times larger than that recorded in the tibia sample (^87^Sr/^86^Sr_A–C_ = 0.00010). Isotopic differences between the different bone tissues were also observed in the Pb isotope data. The average difference between in “A” and “C” samples for the ^206, 208^Pb/^204^Pb and ^207, 208^Pb/^206^Pb for the iliac samples were 0.37, 0.28, 0.01, and 0.03, respectively. In contrast, the average differences for the same ratios in the tibia samples were significantly lower, 0.06, 0.05, 0.001, and 0.003, respectively. Moreover, the Pb isotope composition of all iliac “C” samples clearly shifted towards the “local” Pb isotope signature. In contrast, the Pb isotope composition in the tibia “C” samples shifted in the opposite direction, away from the local signature, except for the open pit burials, donations 1 and 4, and the ^206^Pb/^204^Pb of donation 5. Although natural intra-bone variation cannot be excluded, the data indicate a significantly greater effect of diagenesis on the Sr–Pb isotope composition of the trabecular bone samples compared to the cortical bone samples.

In addition, the observed post-burial changes in Sr isotope composition are more significant in the bone samples from the donations placed in an open pit (donations 1 and 4—open pit burials, average ^87^Sr/^86^Sr_A–C_ in the iliac bone = 0.0010, and tibia bone = 0.0001) compared to the burials (donations 2 and 3—closed burials, average ^87^Sr/^86^Sr_A–C_ in the iliac bone = 0.00010, and tibia bone = 0.00002). Moreover, in both open pit placements, the ^206^Pb/^204^Pb of the iliac “C” samples changed to an isotopic ratio compatible with the local environment (^206^Pb/^204^Pb_soil_ = 19.13–19.44). This is also observed in the ^207^Pb/^206^Pb and ^208^Pb/^204^Pb in open pit donations 1 and 4, respectively (Supplementary Information 2—SI2). Consequently, besides bone type, the mode of body placement also appears to influence ^87^Sr/^86^Sr.

The significant isotopic difference in Sr isotope ratios observed between the “A” and “C” iliac samples in donation 4 (^87^Sr/^86^Sr_A–C_ = 0.001), is consistent with diagenetic alteration of the bone, which is likely related to the long duration of exposure to environmental factors (open pit burial for 872 days). This iliac sample exhibits the lowest “A” ^87^Sr/^86^Sr (0.7084), thus the largest isotopic difference between the bone and the soil. An increase in [Sr] from 47 ppm (“A”) to 90 ppm (“C”, see Table 2). Such significant increases in [Sr] are not observed in any of the other bone samples.

#### Dental elements

The differences in Sr–Pb isotope compositions in the dental enamel are within analytical error (2SE), except for donation 1 (see below). The observed isotopic differences in donation 1, most striking in the ^208^Pb/^204^Pb data (^208^Pb/^204^Pb_A–C_ = 0.036, almost 15 times the analytical error) are explained by the fact that a canine (FDI 43) instead of a lateral incisor (FDI 32) was available as a post-burial “C” sample. Canine enamel mineralises at slightly different ages (4 months–7 years in canines vs. 10 months–5 years in lateral incisors [16]), thus representing a different period in life. The “A” and “C” isotope data are therefore incomparable. The difference in Sr isotope composition between the “A” and “C” dentine samples is negligible, even for donation 1 (^87^Sr/^86^Sr_A–C_ ≤ 0.00008). Although isotopic differences in the dental enamel exceeding the analytical error are observed (e.g. donation 3 ^208^Pb/^204^Pb_A–C_ = 0.028), none of the Pb isotope ratios of the post-burial “C” samples shifted towards the local isotopic signature. The isotopic differences are therefore most likely ascribed to natural intra-dental elemental variation.

## Conclusion and forensic implications

Isotope analysis is a valuable tool in forensic casework [43]. However, in cases where the unidentified remains have been buried or exposed to the weather, the interpretation of isotopic data is not straightforward. This study examined various factors that potentially influence pre- and post-placement Sr–Pb isotope compositions, including the tissue type (keratin vs. bone vs. dental element), the type of bone (trabecular vs. cortical), the skeletal element (long bone, irregular bone, etc.), time since placement outdoors, environmental and climatological conditions during outdoor decomposition, mode of placement (exposed or buried), initial concentration of Sr–Pb, and the isotopic signature of the local soil.

Our findings indicate that the mode of placement (exposed or buried) can influence the Sr–Pb isotope signatures of a cadaver, depending on the type of tissue analysed. Body donations placed in open pits show the greatest differences between pre- and post-placement isotopic signatures. However, since post-placement data are strongly influenced by local environmental and climatic conditions, it remains difficult to extrapolate these results to other regions without careful consideration of the local environment and climate.

In addition, some of the isotope changes recorded in this small-scale study could potentially reflect natural variation (biogenic signature), a diagenetic signature, or a combination thereof. At this point in time, it is not possible to reliably distinguish the cause of the observed alterations. Further research into the natural variation in Sr–Pb isotope composition of different types of human tissue is necessary to improve the applicability of the Sr–Pb isotope systems in forensic contexts and to better understand diagenetic alteration.

Nevertheless, the results of this study show that mid-diaphysis cortical bone (e.g. tibia, humerus, and femur) and dental elements (enamel and dentine) are good targets for forensic isotope analyses due to their greater resistance to diagenetic alterations. By contrast, spongy bones and hair appear to be poor targets for estimation of the geographic origin, based on the marked variability in Sr–Pb ratios observed in the present study. The usage of scalp hair for estimation of geographic origin is further complexed by the fact that, besides diagenetic alteration of the Sr–Pb isotope composition, the applied cleaning method to remove exogenous Sr–Pb from samples also has a significant effect on, at least, the Sr isotope composition [28, 42, 44]. These findings demonstrate that tissue sampling strategies must consider distinct differences in the responses of different human tissues to environment and climatic, as well as sample processing procedures. As such, the results of this pilot study will help to develop sampling strategies that take into account the different diagenetic susceptibility of different human tissues in medicolegal casework. Importantly, our results also emphasise the pressing need for further experimental studies in other locations, as well as the increase in sample size for future studies.

### Supplementary Information

Below is the link to the electronic supplementary material.

Supplementary file1 (DOCX 17 KB)

Supplementary file2 (XLSX 37 KB)

## Data Availability

The data that support the findings of this study are openly available in IsoArcH (www.isoarch.eu; Salesse et al. 2018).
