# Supplementary material for: Sr–Pb isotope differences in pre- and post-burial human bone, teeth, and hair keratin: implications for isotope forensics
Source: Int J Legal Med. 2023 Feb 23;138(1):151–64. doi: 10.1007/s00414-023-02976-5 (PMC10772009; doi:10.1007/s00414-023-02976-5)
Supplement: Supplementary file 1 — Supplementary file1 (DOCX 17 KB) [file 414_2023_2976_MOESM1_ESM.docx]

**Supplementary Information 1**

Sr-Pb isotope differences in pre- and post-burial human bone, teeth, and hair keratin: Implications for isotope forensics

Lisette M. Kootker, Saskia T.M. Ammer, Daniel J. Wescott, Gareth R. Davies, Hayley L. Mickleburgh

**Sample preparation**

All sample preparations were executed at the Vrije Universiteit Amsterdam. Depending on the task, the work was performed in either the semi-clean Archaeological and Forensic sample preparation laboratory or the US Federal Standard Class (FED-STD-209E) 100 clean laboratory facility (ISO 5), equipped with FED-STD Class 10 flow hoods (ISO 4).

*Soil*

The soil samples were dried overnight on a hotplate at 60 ºC, gently stirred with mortar and pestle to loosen the soil particles and to homogenise the sample. Sub-samples of circa 30 grams were placed in glass beakers. The samples were sieved over a stainless steel 2 mm sieve (ISO 3310) and 2 g of the <2 mm fraction was transferred to acid pre-cleaned centrifuge tubes. To extract the soil’s mobile, water-soluble, and bioavailable Sr, the DIN ISO 19730:2009-07 protocol was followed. The sub-sample was leached by adding 5 mL 1M ammonium nitrate (NH_4_NO_3_) and placed on a rocker for 8 hours. The samples were then transferred to the clean laboratory facility where they were centrifuged for 15 minutes at 3000 RMP. The supernatant was extracted and collected in 7 mL acid pre-cleaned PFA vials, evaporated to dryness and subsequently re-dissolved in 500 μL 3M HNO_3_ for Sr purification.

*Scalp hair*

The pre-treatment protocol used for the hair samples is explained in detail in Kootker *et al.* [28]. In short, the hair samples were cleaned with high pressure N_2_ gas to remove adhered soil and dust particles. Hair was then washed with Milli-Q and a 2:1 chloroform/methanol mixture. For consistency and comparison purposes, an identical protocol was applied to the hair samples from donation 5. Once transferred to the clean laboratory facility, the hair samples were dissolved following the bone protocol outlined below and ICP-MS aliquots were taken. Remaining samples were dried and re-dissolved in 500 μL 0.7M HBr for Sr-Pb separation.

*Teeth*

The dental elements were cleaned with Milli-Q water and a toothbrush to remove adhering particles. The enamel surface and a root were cleaned with an acid-cleaned diamond-tipped burr. For Sr-Pb isotope analyses, approximately 30 mg of enamel powder was collected in clean glass vials. A root was sampled using a Dremel with a diamond wheel drill piece and collected in an acid-cleaned Eppendorf®. Both sample types were then transferred to the clean laboratory facility. The samples were weighed into pre-cleaned Teflon beakers and dissolved in HCl (6-7M) and concentrated HNO_3_ (14M). Once dried on a hotplate overnight at 120 ºC, the samples were dissolved in 500 μL 3M HNO_3_ (enamel). A known aliquot volume was analysed using ICP-MS to determine the Sr-Pb concentrations. The remaining samples were dried and re-dissolved in 500 μL 0.7M HBr for Pb-Sr separation. The dentine samples followed the same steps as the bone samples (section 3.3.4).

*Bone*

The bone samples were cut using a Dremel with a diamond wheel drill piece into three sub-samples for Pb-Sr isotope analysis (N=1) and future research (N=2). The sample weights ranged from 30 to 150 mg. The outer surface of the bone samples was mechanically cleaned with an acid-cleaned diamond-tipped burr, and further treated overnight with a 2:1 chloroform/methanol mixture on a rocker to induce chemical lipid extraction. The samples were rinsed three times with Milli-Q water and left to dry on a hotplate at 50 ºC. Once dry, the samples were transferred to the clean laboratory facility and weighed into pre-cleaned Teflon beakers. The samples were dissolved in a mixture of H_2_O_2_ (30%, ultrapure), HCl (6-7M) and concentrated HNO_3_ (14M), and subsequently dried on a hotplate at 120 ºC. This step was repeated until all organics were removed. Once samples were fully dissolved, they were dried and re-dissolved in 500 μL 3M HNO_3_. A known aliquot volume was taken for ICP-MS analysis to determine the Sr-Pb concentrations. The samples were then dried and taken up in 500 μL 0.7M HBr for Sr-Pb isotope separation.
